# Theta phase coherence in visual mismatch responses involved in access processing to visual awareness

**DOI:** 10.3389/fnhum.2023.1051844

**Published:** 2023-02-23

**Authors:** Yuki Kurita, Tomokazu Urakawa, Osamu Araki

**Affiliations:** ^1^Department of Applied Physics, Faculty of Science, Tokyo University of Science, Tokyo, Japan; ^2^Integrated Control System Development Division, Mazda Motor Corporation, Hiroshima, Japan

**Keywords:** visual mismatch oscillatory responses (vMORs), visual mismatch negativity (vMMN), binocular rivalry, time frequency representation (TFR), unconsciousness, perceptual alternation

## Abstract

**Introduction:** The electroencephalographic brain response to a deviation from the preceding sequential regularity of visual events, called visual mismatch negativity (vMMN), is well known to reflect automatic visual change detection. Our preliminary study showed a significant correlation between the enhancement of the vMMN amplitude and facilitation of perceptual alternation in binocular rivalry when the deviant stimulus was presented unconsciously. This implies that the vMMN is relevant to access processing, in which the unconscious stimulus is consciously perceived. Recent studies have reported that theta band oscillation evoked by a deviant stimulus is involved in evoking vMMN. However, it has not been clarified whether theta band oscillation associated with vMMN is also relevant to access processing.

**Methods:** We analyzed the correlations between event-related spectral perturbation (ERSP) and inter-trial phase coherence (ITPC) in the theta band and the proportion of perceptual alternation from before to after the presentation of deviation in the same experimental paradigm as in our previous study.

**Results:** We found that an increase in ITPC in the theta band tended to correlate with facilitation of perceptual alternation in binocular rivalry when the deviant was presented unconsciously, but there was no significant correlation in ERSP.

**Discussion:** The results suggest that theta phase coherence underlying the visual mismatch process is relevant to the access processing.

## Introduction

Early neural mechanisms related to conscious experience in vision have been investigated by comparing neural responses (e.g., time-frequency representation) when the visual stimulus is consciously perceived and when it is not consciously perceived (Hanslmayr et al., 2007; Melloni et al., 2007; Wyart and Tallon-Baudry, 2008; Doesburg et al., 2009; Aru et al., 2012; Lange et al., 2013). Previous studies focusing on oscillatory responses indicated that an increase in gamma band activity and a decrease in alpha band activity were observed when the visual stimulus was consciously perceived (for review, see Ceylan et al., 2016; Koch et al., 2016; Gallotto et al., 2017). However, oscillatory responses related to unconscious neural processing have not yet been clarified. Recent studies have reported that theta band activity in the posterior and frontal cortical areas is also involved in conscious visual perception (Davidson et al., 2018; Drew et al., 2019; Haque et al., 2020). In particular, Haque et al. (2020) reported that each mean amplitude of theta oscillations in occipital, posterior parietal, and prefrontal cortices correlates with the intensity of the conscious experience (e.g., visibility ratings: “Not visible,” “Barely,” “Partially,” and “Fully”). This correlation suggests that theta activity reflects the steps toward full consciousness in neural mechanisms (Haque et al., 2020). In other words, theta activity appears to be involved in unconscious neural processing [Hereafter, access processing to visual awareness (APVA)] that determines whether the unconscious visual stimulus is consciously perceived.

In the perceptual process of binocular rivalry, which is often used in studies on visual awareness, the involvement of theta activity has been reported as follows. Inter-trial phase coherence (ITPC) at 3.5 Hz in the fronto-temporal area and at 8 Hz in the parieto-occipital area correlated with changes in conscious perception (Davidson et al., 2018). Oscillatory power at 5–7 Hz in the fronto-medial area is also related to the resolution of perceptual conflict (Drew et al., 2019, 2022). According to these results, theta band neural activity (4–8 Hz) is thought to be involved in shaping a conscious perception in binocular rivalry.

In contrast, a previous study reported that visual mismatch negativity (vMMN) is relevant to APVA (Kurita et al., 2021). VMMN is known to have a negative-going neural component over posterior electrodes at a latency of approximately 130–250 ms when comparing responses to an infrequently presented stimulus (deviant) and a repetitively presented stimulus (standard; Czigler et al., 2002; Astikainen et al., 2008; Kimura et al., 2009). This negativity has been interpreted to reflect automatic visual change detection based on temporal regularity. To clarify neural processing before visual stimuli are consciously perceived, Kurita et al. (2021) focused on the vMMN as one of the earliest brain activities related to visual awareness. They presented target stimuli (standard and deviant) consciously or unconsciously using binocular rivalry and then observed the proportion of perceptual alternation from before to after the onset of the target stimulus and electroencephalography (EEG) signals. The results of the experiment showed a positive correlation between the enhancement of vMMN amplitude and the facilitation of perceptual alternation when the deviant was presented unconsciously. However, the previous study did not evaluate induced responses such as oscillatory activities but only neural activity time-locked to the stimulus onset. On this point, our previous study did not disclose a full picture of visual mismatch processing underlying APVA.

As described above, the theta band neural activity and vMMN have been independently reported to be involved in APVA. In contrast, oscillatory responses related to the visual mismatch process (visual mismatch oscillatory responses, vMORs) have been reported to appear in the theta band (Stothart and Kazanina, 2013; Hesse et al., 2017; Yan et al., 2017), suggesting that these neural activities are not independent of each other. According to these studies, vMORs enhance the oscillatory response to the deviant stimulus relative to the standard stimulus over the posterior electrodes at a latency of approximately 100–350 ms. They are interpreted to reflect automatic visual change detection or memory-based comparison of the preceding visual stimuli. In addition, event-related spectral perturbation (ERSP) in theta band vMORs is considered to be involved in the generation of vMMN (Stothart and Kazanina, 2013; Yan et al., 2017). This indicates that oscillatory responses in the theta band are related to the neural mechanism of the visual mismatch process. If theta band activity is related to the cognitive process of conscious perception as described in the first paragraph, vMORs in the theta band will also play an important role in APVA. Therefore, we hypothesized that vMORs in the theta band are involved in the neural mechanisms of APVA.

The purpose of the present study is to examine the relationship between theta band vMORs and APVA. To clarify whether vMORs in the theta band affect APVA, we further analyzed ERSP and inter-trial phase coherence (ITPC) in the theta band for EEG data in our previous study (Kurita et al., 2021). We then investigated the inter-individual variability between ERSP/ITPC enhancement in the theta band and the proportion of perceptual alternations. The present study is expected to clarify whether neural processing related to vMORs, which is little known so far, is involved in APVA, and if so, how it is related.

In the present study, while the experimental paradigm, participants, and the behavioral and recorded EEG data are common to our previous study (Kurita et al., 2021), analyses such as ERSP and ITPC were additionally executed to reach the goal.

## Materials and methods

### Participants

All participants in this study are the same as those in our previous study (Kurita et al., 2021). Nineteen healthy volunteers (19 males, aged 21–36 years, mean ± SD, 23.2 ± 0.76 years) participated in this experiment. All the participants were right-handed and had normal or corrected-to-normal visual acuity. Informed consent was obtained from all participants, and this study was approved by the ethics committee of Tokyo University of Science.

### Stimulus and procedure

**Figure 1:**
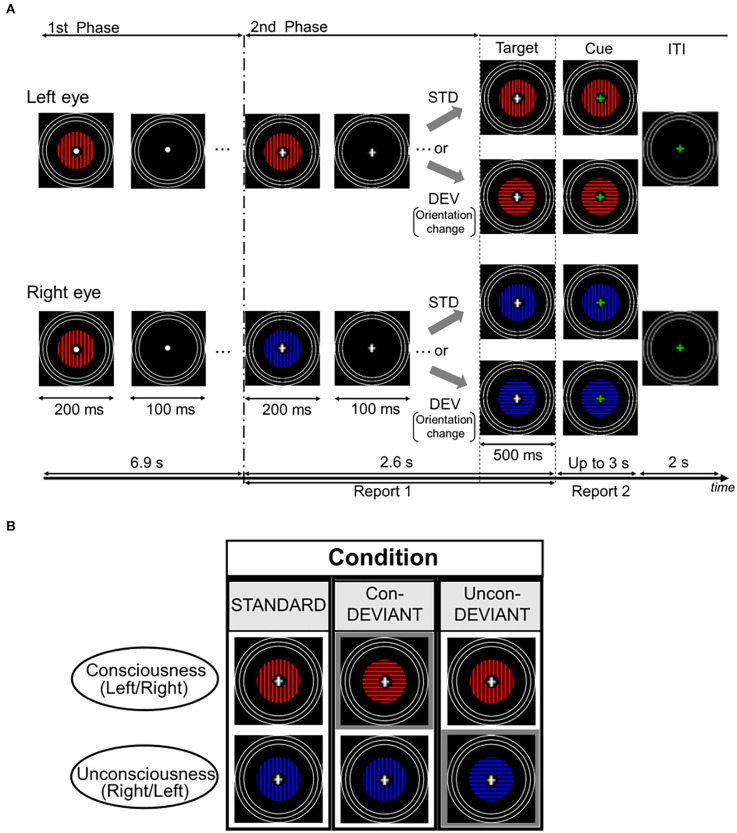
Time course of stimulus presentation in one trial and experimental conditions. **(A)** Each trial consisted of two stimulation phases. In the first phase, an identical grating stimulus with a color (blue or red) was simultaneously and intermittently presented for both the left eye and the right eye. Participants were asked to passively look at the fixation point. In the second phase, a color of the grating stimulus changed for either of the two eyes to induce the binocular rivalry (a color change from blue to red or vice versa), and the grating images were presented intermittently as in the first phase. During this phase, the fixation cross was continuously presented at the central area of the gratings, and participants were required to continuously report the perceived grating. Following the second phase, the target stimulus immediately appeared for 500 ms and its fixation cross then changed in color to a green cross (the cue). Participants were asked to report the current perceived grating stimulus after the onset of the cue. **(B)** Each pair of two grating stimuli arranged vertically indicates an example of the target stimulus. Target stimulus had three variants by changing or not changing an orientation of the grating stimulus under the binocular rivalry. In the Uncon-DEV condition, an orientation of the grating stimulus which did not reach a conscious percept was exclusively changed by 90°. Meanwhile, in the Con-DEV condition, the orientation of the grating stimulus that reached a conscious percept was exclusively changed by 90°. In the STD condition, there was no change in the grating stimulus for both eyes. Figure 1 in our previous study (Kurita et al., 2021) has been reproduced here because of the same experimental procedure. STD, standard; Con-DEV, conscious-deviant.

Figure 1 shows the stimuli and the stimulation procedure for one trial. Images were presented on a liquid crystal display (BenQ XL2540) using the MATLAB Psychophysics Toolbox (Brainard, 1997; Pelli, 1997). The participants were presented with two computer-generated images using a mirror stereoscope. The image included annulus-shaped gratings with a spatial frequency of 1.3 cycles/degree. The outer radius of the gratings was 4.3° and the inner radius was 0.57°. A white fixation point was presented at the center of the grating image. The blue or red grating was presented on a black background, with a mean luminance of 0.05 cd/m^2^. The mean luminance of the red portion was 3.56 cd/m^2^, whereas that of the blue portion was 2.16 cd/m^2^. Each grating was surrounded by three white rings that served to lock the vergence. Each white ring had a line width of 0.19°. The outer radius of the largest ring was 8.64°, and the outer edges of each of the other two smaller rings were inwardly depicted by 0.64° from the outer edge of the neighboring larger ring. White rings in both eyes were continuously presented throughout the stimulation period.

Each trial consisted of two consecutive phases (Figure 1A). In the first phase, an identical grating image was simultaneously presented to both the left and right eyes. The grating images were intermittently presented 23 times, with a duration and inter-stimulus interval (ISI) of 200 ms and 100 ms, respectively. The grating was either blue or red, and its orientation was either horizontal or vertical. Between these images, an image without grating was presented for 100 ms in both eyes. In the presentation of the grating image, the colors were counterbalanced across the trials for each participant. The orientation of the grating in the first phase was maintained constant for each participant, and then it was counterbalanced among the participants. In the first phase, participants were asked to look at the fixation point passively. Immediately following the first phase, in the second phase, the grating image was manipulated to induce binocular rivalry by changing its color from blue to red or *vice versa* for either eye at the beginning of the second phase. The color change was counterbalanced between the two eyes. Similar to the first phase, the gratings in the second phase were simultaneously and intermittently presented, without changing the grating image for each eye. The duration and ISI used in the second phase were the same as those used in the first phase. They were expected to mitigate binocular fusion (Wolfe, 1983). According to the previous psychophysiological studies (e.g., Astikainen et al., 2008; Kimura et al., 2009; Urakawa et al., 2017, 2018), vMMN was also expected to appear in the duration and ISI. In the intermittent stimulation of the second phase, the gratings were presented seven times. During the second phase, a white fixation cross appeared at the center of the grating image instead of the white fixation point. When the white cross appeared (i.e., when the second phase started), the participants were required to fixate on the cross and press a key on the keyboard in front of them to start reporting a perceived grating image. In this behavioral task, participants were asked to continuously press the left arrow key during the period in which blue grating was perceived or to continuously press the right arrow key during the period in which red grating was perceived. Meanwhile, during the period in which blue and red gratings merged in perception, the participants were instructed not to press any key. This behavioral task was continued throughout the second phase. In every trial, participants were asked to maintain their initial perception during the second phase as much as possible. The second phase ended with a blank image that lasted for 100 ms, as in the first phase. Following the termination of the second phase, the target stimulus was immediately presented for 500 ms. The change in orientation (from horizontal to vertical or vice versa) corresponded to the deviant that violated the preceding sequential regularity, which was a repetition of an identical orientation from the beginning in the first phase. In this orientation manipulation, the colors of both eyes remained the same. As illustrated in Figure 1B, the target stimulus yielded three conditions dependent on the subject’s conscious/unconscious percept just before itself: the standard (STD) condition, the unconscious-deviant (Uncon-DEV) condition, and the conscious-deviant (Con-DEV) condition. In the STD condition, the target stimulus was the same as the grating images used in the second phase, except for the duration (no change in orientation). In the Uncon-DEV condition, the orientation of the grating, which appeared “unconsciously,” changed by 90°. In the Con-DEV condition, the orientation of the grating, which was perceived “consciously,” was changed by 90°. Stimuli presented to both eyes in each trial were determined based on the perceptual report immediately prior to the target stimulus. The target stimulus was immediately followed by a cue image, which appeared for up to 3 s. In the cue image, the white fixation cross of the target stimulus was replaced with a green fixation cross for both eyes. When green fixation appeared, the participants were asked to stop pressing the left arrow key or the right arrow key immediately. They were then required to promptly report their currently perceived grating image again by pressing either the left arrow key or the right arrow key, as in the task during the second phase. Upon pressing the key, the cue image disappeared. The inter-trial interval (ITI) was 2 s. During ITI, rings, and green fixation points were exclusively presented. Each of the three conditions contained 120 trials. The order of these stimulus conditions was randomized across trials. There were eight sessions in the present study, each of which had 45 trials. Participants were given rest between sessions, as needed.

### Analysis of behavioral data

Analytical methods of behavioral data are common to our previous study (Kurita et al., 2021). Owing to the latency of the behavioral response, the timing of the participants’ keypress would lag from the perceived rivalry changes by approximately 450–500 ms (Alais et al., 2010). In this analysis, we first counted the trials in which participants pressed a response key for at least 500 ms immediately before the onset of the target stimulus. We then subtracted the number of trials in which a participant did not stop pressing the key or did not press it again following the onset of the cue image. In this procedure, trials in which participants responded within 300 ms of the cue onset were also excluded to ensure that participants had correctly checked the cue. These procedures enabled us to obtain valid trials and count the number of times when the perceived color changed from before to after the onset of the target stimulus, and then calculated the proportion of perceptual alternation for every condition.

### EEG recording

Electroencephalography (EEG) in each condition was recorded using a measurement instrument with 57 electrodes (EEG-1200, Nihon Kohden, Tokyo, Japan; EasyCap GmbH, Herrsching, Germany). The layout of the electrodes was based on a modified version of the international 10–20 system. The impedance of each electrode was maintained at less than 10 kΩ. EEG signals were digitized at 1 kHz and recorded with a 0.5–300 Hz band-pass filter online. For data acquisition, EEG signals were referenced to the right earlobe and eye movements were monitored using horizontal and vertical bipolar electrooculograms (EOGs).

### Analysis of EEG data

EEG epochs from 400 ms before to 600 ms after the onset of the target stimulus in valid trials were collected (see Section “Analysis of behavioral data” for details). EEG epochs containing a deflection greater than ± 100 μV in at least one electrode or greater than 100 μV in EOGs were excluded from this analysis. With this procedure, at least 77 artifact-free EEG epochs (mean ± SD, STD condition: 105.2 ± 11.8 trials, Con-DEV condition: 104.6 ± 13.0 trials, Uncon-DEV condition: 104.9 ± 8.7 trials) were obtained. These epochs were sorted according to the target stimulus conditions and then transformed into time-frequency representations *via* a complex Morlet wavelet transformation using the MATLAB wavelet toolbox. The mother cycles were set to a linear increase of 2–7 cycles with respect to the frequency range (1–50 Hz). The ERSP and ITPC of each participant were calculated relative to the baseline (−400 to −100 ms) for each electrode. To record vMORs evoked by the unconscious deviant (Uncon-vMORs) as well as those evoked by the conscious deviant (Con-vMORs), ERSP and ITPC in the STD condition were subtracted from those in the Uncon-DEV and Con-DEV conditions, respectively. For both Con-vMORs and Uncon-vMORs, the ERSP and ITPC in the left area were calculated from the mean value of data PO3 and PO7 electrodes, and these in the right area were calculated from the mean value of data PO4 and PO8 electrodes. And then, we calculated the averaged ERSPs and ITPCs for each participant in the time-frequency window of 100–500 ms and 4–8 Hz, respectively. This time-frequency window was determined in previous studies on vMORs (Stothart and Kazanina, 2013; Yan et al., 2017). The calculated ERSPs and ITPCs were subjected to a repeated-measures two-way analysis of variance (ANOVA) with factors of the conditions (Con-vMOR and Uncon-vMOR) and laterality (left and right areas), respectively. In the statistical analyses, the significance level was set at *p* < 0.05.

### Correlation analysis

As in a previous study (Kurita et al., 2021), we further performed correlation analyses between the differential proportion of perceptual alternation and ERSP/ITPC of vMORs (Con-vMORs or Uncon-vMORs) across participants. The differential proportion was calculated by subtracting the proportions of perceptual alternation between the conditions; Con-DEV and STD, or Uncon-DEV and STD. Inter-individual differences of obtained data were all collapsed in the ANOVA results while correlation analyses were performed in terms of the inter-individual differences of behavioral and neural data. The correlation analysis enabled us to discover a relationship which was hidden in averaging over participants. Such correlation analyses between behavioral indices and neural data, with a focus on inter-individual differences, are expected to be a powerful approach in deducing the neural mechanisms underlying behavioral data (e.g., Vogel and Awh, 2008). The Spearman’s rank-order correlation coefficients were calculated. In the analyses, the significance level was set at *p* < 0.05.

## Results

### Behavioral data

The results of behavioral data are common to our previous study (Kurita et al., 2021). Those results indicate that the unconscious deviant facilitates the proportion of perceptual alternation (Walker and Powell, 1979). In contrast, the conscious deviant suppresses the proportion of perceptual alternation (for more detailed statistical results, see Kurita et al., 2021).

### EEG data

**Figure 2:**
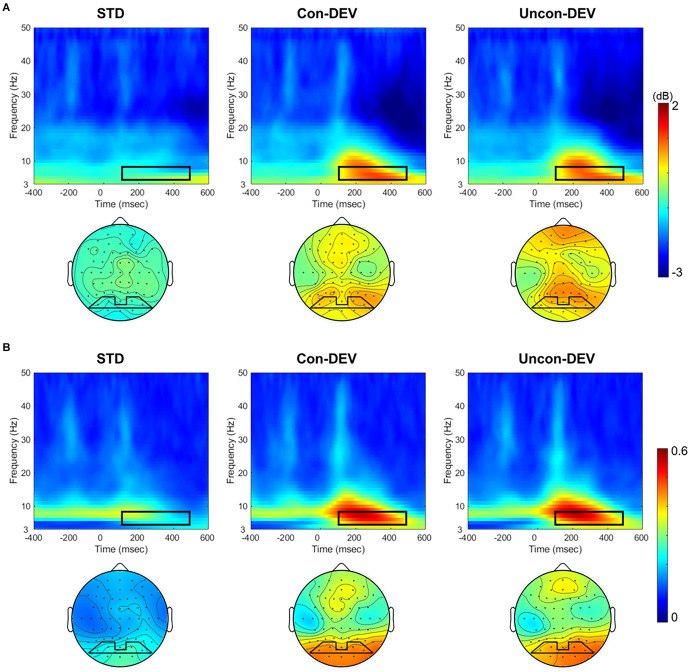
ERSP and ITPC to the target image for each target condition. ERSPs **(A)** and ITPCs **(B)** at the posterior area (PO3, PO7, PO4, and PO8) and their isocontour maps averaged from 4 to 8 Hz at 100 to 500 ms are illustrated for each condition. ERSPs and ITPCs in both the Con-DEV and Uncon-DEV conditions were more enhanced than those in the STD condition in the time-frequency windows of 100–500 ms and 4–8 Hz. ERSP, event-related spectral perturbation; ITPC, inter-trial phase coherence.

Figure 2 shows the averaged ERSP and ITPC in the posterior area (PO3, PO7, PO4, PO8) and the averaged isocontour map in the time-frequency window for each condition. ERSP and ITPC in the theta band appear to be enhanced in approximately 100–500 ms time window for the Con-DEV condition or the Uncon-DEV condition than for the STD condition in the posterior area. These results are consistent with those of previous studies on vMORs (Stothart and Kazanina, 2013; Yan et al., 2017).

**Figure 3:**
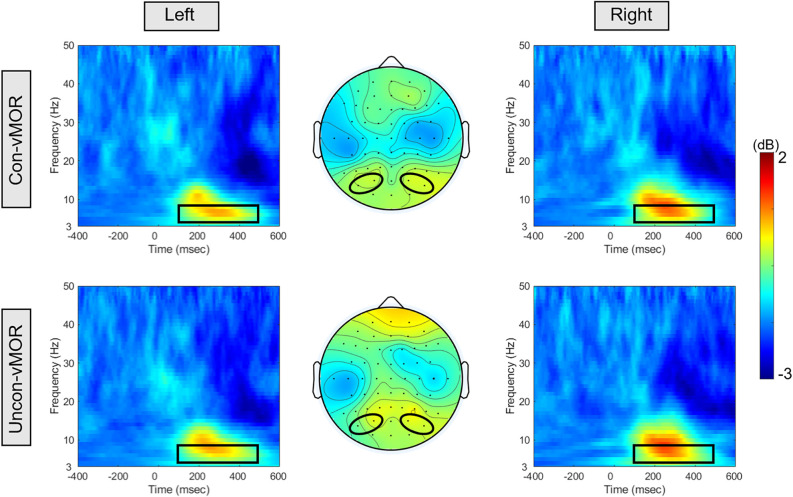
ERSPs and their isocontour map for each condition. The panels show the ERSPs in the Con-vMOR condition in the left area (PO3, PO7; upper left panel), the Con-vMOR condition in the right area (PO4, PO8; upper right panel), the Uncon-vMOR condition in the left area (lower left panel), the Uncon-vMOR condition in the right area (lower right panel), and their isocontour maps (the upper map is in the Con-vMOR condition, and the lower map is in the Uncon-vMOR condition). The black box in the ERSPs indicates a time-frequency window of 100–500 ms and 4–8 Hz. Con-vMOR, conscious-visual mismatch oscillatory response; Uncon-vMOR, unconscious-visual mismatch oscillatory response.

Figure 3 shows the ERSPs of Con-vMORs and Uncon-vMORs in the right and left areas, respectively, and the averaged isocontour map in the time-frequency window. An increase in ERSP was observed at 6–8 Hz between approximately 200–400 ms, for both the Con-vMOR and the Uncon-vMOR. A repeated-measures two-way ANOVA revealed that the enhancement of ERSP was not significantly affected by the condition and laterality (Condition: *F*_(1, 18)_ = 0.003, *p* = 0.956, partial *η*^2^ < 0.01; Laterality: *F*_(1, 18)_ = 0.245, *p* = 0.626, partial *η*^2^ = 0.013), or interaction (*F*_(1, 18)_ = 0.429, *p* = 0.521, partial *η*^2^ = 0.023). These results indicated that the increase of theta band ERSP by the deviant stimulus is independent of whether the deviant stimulus is presented consciously or unconsciously, and that there is no significant difference in the increase of ERSP between the left and right areas.

**Figure 4:**
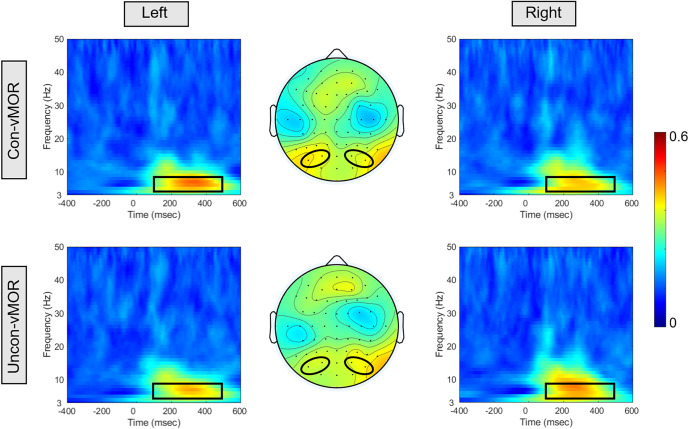
ITPCs and their isocontour map for each condition. The panels show the ITPCs in the Con-vMOR condition in the left area (PO3, PO7; upper left panel), the Con-vMOR condition in the right area (PO4, PO8; upper right panel), the Uncon-vMOR condition in the left area (lower left panel), the Uncon-vMOR condition in the right area (lower right panel), and their isocontour maps (the upper map is in the Con-vMOR condition, and the lower map is in the Uncon-vMOR condition). The black box in the ITPCs indicates a time-frequency window of 100–500 ms and 4–8 Hz.

Figure 4 shows ITPCs of Con-vMORs and Uncon-vMORs in the right and left areas, respectively, and the averaged isocontour map in the time-frequency window. An increase in ITPC was observed at 6–8 Hz between approximately 200–400 ms for both the Con-vMOR and the Uncon-vMOR. A repeated-measures two-way ANOVA revealed that the enhancement of ITPC was not significantly affected by the condition or laterality (Condition:* F*_(1, 18)_ = 0.377, *p* = 0.547, partial *η*^2^ = 0.021; Laterality: *F*_(1, 18)_ < 0.001, *p* = 0.985, partial *η*^2^ < 0.01), and that the enhancement of ITPC was significantly affected by the interaction between the condition and the laterality (*F*_(1, 18)_ = 14.695, *p* < 0.01, partial *η*^2^ = 0.449). An analysis of multiple comparisons further revealed that the ITPC in the left area under the Uncon-vMOR condition was marginally significantly lower than that under the Con-vMOR condition (Condition in Left: *F*_(1, 18)_ = 3.211, *p* = 0.090, partial *η*^2^ = 0.151, a *post-hoc* test with a Bonferroni correction), and the other simple main effects were not significant (Condition in Right: *F*_(1, 18)_ = 0.754, *p* = 0.397, partial *η*^2^ = 0.040, a *post-hoc* test with a Bonferroni correction; Laterality in Con-vMOR: *F*_(1, 18)_ = 0.488, *p* = 0.494, partial *η*^2^ = 0.026, a *post-hoc* test with a Bonferroni correction; Laterality in Uncon-vMOR: *F*_(1, 18)_ = 0.619, *p* = 0.442, partial *η*^2^ = 0.033, a *post-hoc* test with a Bonferroni correction). These results indicate that the increase of theta band ITPC in the left area by the deviant stimulus tends to differ depending on whether the deviant stimulus is presented consciously or unconsciously.

### Correlation between behavioral data and vMORs

**Figure 5:**
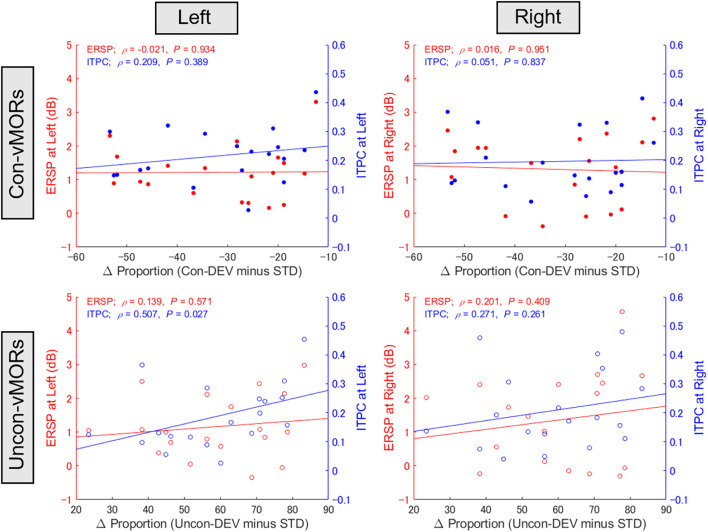
Relationship between proportion of perceptual alternation and vMORs across participants. The correlations between the differential proportion of perceptual alternation (Uncon-DEV condition − STD condition; Con-DEV condition − STD condition) and ERSP/ITPC of vMORs in the theta band are shown for each condition (Con-vMOR in left: upper left panel; Con-vMOR in right: upper right panel; Uncon-vMOR in left: lower left panel; Uncon-vMOR in right: lower right panel). There was a significant correlation between the differential proportion and enhancement of ITPC in the left area under the Uncon-vMOR condition. Uncon-DEV, unconscious-deviant.

Focusing on the inter-individual variability in behavioral data and ERSP/ITPC in the left and right areas under the Con-vMOR and Uncon-vMOR conditions, respectively, we evaluated whether the ERSP/ITPC of vMORs in the theta band would be relevant to the facilitation or suppression of perceptual alternation. Figure 5 shows the results of the correlation analysis for ERSP and ITPC. For ERSP, there was no significant correlation between the differential proportion of perceptual alternation and ERSP in either condition (Con-vMOR on the left: *ρ*_(19)_ = −0.021, *p* = 0.934; Con-vMOR on the right: *ρ*_(19)_ = 0.016, *p* = 0.951; Uncon-vMOR on the left: *ρ*_(19)_ = 0.139, *p* = 0.571; Uncon-vMOR on the right: *ρ*_(19)_ = 0.201, *p* = 0.409). We analyzed the four correlations between ITPC and the differential proportion of perceptual alternation (Con-vMOR on the left: *ρ*_(19)_ = 0.209, *p* = 0.389; Con-vMOR on the right: *ρ*_(19)_ = 0.051, *p* = 0.837; Uncon-vMOR on the left: *ρ*_(19)_ = 0.507, *p* = 0.027; Uncon-vMOR on the right: *ρ*_(19)_ = 0.271, *p* = 0.261). The correlations for ERSPs and ITPCs were not significant after correcting for multiple tests for eight correlations (adjusted* p* < 0.05/8 = 0.00625 with a Bonferroni correction). However, we assume that the data under the conscious and unconscious conditions are independent in later comparison because the stimuli are completely different. Further analysis of the equality of variances for ERSPs and ITPCs using Levene’s test showed that the variances of the ERSP and ITPC groups are different (*W*_(1, 150)_ = 125.8, *p* < 0.001). Thus, we assume that ERSPs and ITPCs are independent in the later comparison. Taken together, in the correlation analyses of ITPC, we corrected for multiple tests for laterality with a Bonferroni correction (adjusted significance level was set at *p* < 0.05/2 = 0.025). As a result, a positive correlation between the differential proportion of perceptual alternation and ITPC in the left area in the Uncon-vMOR condition is marginally significant. These results show that an enhancement of theta band ITPC in the left area by the unconscious deviant stimulus is correlated with the facilitation of perceptual alternation across participants. Considering these results, an increase in the theta band ITPC in the left posterior area is more closely related to rendering an unconsciously presented image perceived consciously than that of ERSP. Note that the correlation between ITPC in the left area and the proportion of perceptual alternation under unconscious conditions was shown based on the assumptions described above.

**Figure 6:**
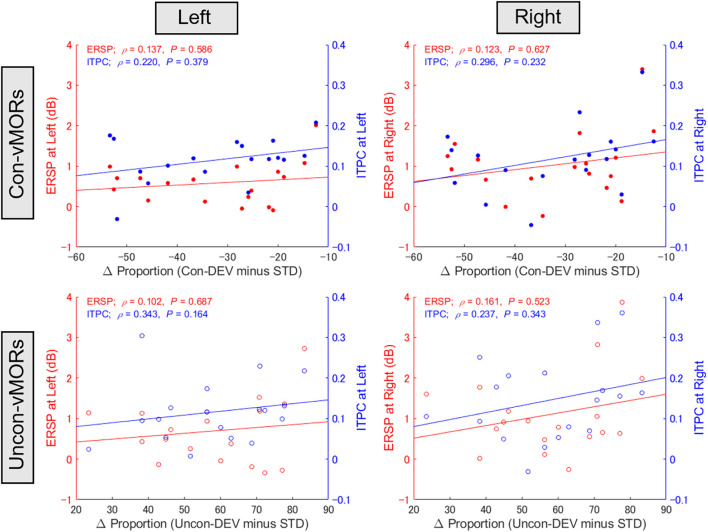
Relationship between proportion of perceptual alternation and alpha-band ERSP/ITPC. The correlations between the differential proportion of perceptual alternation and ERSP/ITPC of vMORs in the alpha band are shown for each condition. ERSP and ITPC in the left area (PO3, PO7) and right area (PO4, PO8) were calculated for each participant in the time-frequency window of 100–500 ms and 9–13 Hz, respectively.

The present study supplementarily evaluated whether the differential proportion of perceptual alternation would be associated with ERSP or ITPC at the alpha-band across participants (see Figure 6). One participant’s data were excluded from this analysis because the data for ERSP at the left hemisphere under conscious condition exceeded the range of the mean ± 3 SD. There was no significant correlation between the differential proportion and alpha-band ERSP/ITPC under any condition.

## Discussion

This study attempted to clarify whether vMORs in the theta band are related to APVA. We presented the deviant stimulus under binocular suppression or dominance and then investigated the relationship between perceptual alternation and enhancement of ERSP or ITPC in the theta band. Consequently, there was no significant correlation between ERSPs and ITPCs after correcting for multiple tests. However, assuming that the data under each condition of Conditions (conscious and unconscious conditions) and Types (ERSPs and ITPCs) are statistically independent, we found a marginally significant positive correlation between the facilitation of perceptual alternation and an increase in ITPC when the deviant stimulus was presented unconsciously. On the other hand, no significant correlation was observed in ERSP when the deviant stimulus was presented unconsciously. These results suggest that phase alignment in the theta band underlying the visual mismatch process is involved in APVA.

In the present study, a weak correlation between perceptual alternation and theta band ITPC in the left posterior area was observed under the Uncon-vMOR condition. According to the ANOVA for ITPC (see “EEG data” section), the posterior ITPC in the left hemisphere under the Uncon-vMOR condition tended to be lower than that under the Con-vMOR condition. Despite the smaller quantity of ITPC, the results showed that the left posterior theta ITPC evoked by unconscious deviant stimuli is only relevant to the facilitation of perceptual alternation. The theta ITPC under the conscious condition was higher than the unconscious one. This tendency is consistent with the results in previous vMMN studies (Jack et al., 2017). A previous study reported that theta oscillations in the left paracentral lobule encoded the resolution of conflicts induced by both stimulus-related conscious and unconscious information processes (Giller et al., 2020). Similarly, in binocular rivalry, theta oscillatory activity in the left hemisphere is considered to be involved in neural processing, which determines conscious perception by solving the conflict between conscious and unconscious information. Therefore, these results indicate that the theta-band posterior ITPC in the left hemisphere is relevant to the unconscious process of determining perceptual alternation in binocular rivalry. It is unclear whether vMMN in either the right hemisphere (Kimura et al., 2009) or bilateral posterior (Urakawa et al., 2017, 2018; Kurita et al., 2021) relates to the correlation in the left ITPC. The left ITPC may mainly reflect higher perceptual functions such as conflict resolution rather than the change detection reflected by vMMN.

Under the assumption that the data under each of the four conditions [Conditions (conscious and unconscious conditions) and Types (ERSPs and ITPCs)] are independent in multiple comparisons of correlation coefficients, we found that the increase in theta ITPC evoked by the unconscious deviant stimulus at the occipital electrodes tended to make it easier for the stimulus to be consciously perceived. Several studies have suggested that neural theta phase coherence in the visual mismatch process reflects information flow through functional connectivity, not only within the occipital sites, where vMMN is evoked by preattentive visual change detection but also between the frontal and other areas, where the attentional mechanism associated with vMMN is involved (Stothart and Kazanina, 2013; MacLean and Ward, 2014; Hedge et al., 2015). From a theoretical viewpoint, top-down prediction as well as bottom-up stimulus information is indispensable for mismatch neural processing (Kimura, 2012; Winkler and Czigler, 2012). Thus, in the present study, theta phase coherence associated with vMMN in the occipital areas may also be related to the attentional mechanism in the prefrontal cortex. On the other hand, exogenous attention to a certain location in the visual field facilitates the visual processing of a subsequent invisible target image presented at the same location and enhances conscious perception (Chica et al., 2010, 2011). In addition, exogenous attention has been reported to promote perceptual alternations in binocular rivalry (Paffen and Van der Stigchel, 2010; Paffen and Alais, 2011). Since the unconscious deviant, which was an external perturbation, made the unconscious stimulus more likely to be consciously perceived in the present study, exogenous attention induced by the unconscious deviant seems to enhance the conscious perception of the visual stimulus presented unconsciously. Taken together, these results suggest that the theta phase coherence evoked by the unconscious deviant stimulus enhanced the attentional mechanism associated with the automatic process of visual change detection, and its enhancement of the attentional mechanism makes it easier for the unconscious stimulus to be consciously perceived. This indicates that theta alignment evoked by the visual mismatch process and the accompanying enhancement of the attentional mechanism play an important role in the neural mechanism of APVA.

To clarify the effects of the mismatch process on APVA, our previous study showed that neural activity time-locked to the deviant stimulus onset correlated promotion of APVA (Kurita et al., 2021). To clearly specify the processing of APVA driven by the mismatch, the present study analyzed induced oscillatory responses and discovered that the theta-band ITPC of vMORs plays an important role in the neural mechanism of APVA, which seems to enhance the attentional mechanism associated with the mismatch process. In addition to our previous findings regarding vMMNs (Kurita et al., 2021), the present study would further shed new light on the neural mechanism underlying APVA in terms of vMORs.

While no significant correlation was found after correcting for multiple tests, the correlation between an increase of ITPC in the left area and the facilitation of perceptual alternation under unconscious condition was slightly effective with the assumption of statistical independence of the data. However, note the limitations described above (see “Correlation between behavioral data and vMORs” section). Thus, this study should be considered to gradually provide trends in the relationship between vMORs and perceptual alternation. Further research and validation are needed to ensure the reliability of this correlation.

## Conclusions

We captured a weak but important relationship between the increase in theta-band ITPC in the left posterior area and facilitation of perceptual alternation in binocular rivalry when the deviant stimulus was presented unconsciously, with hypothesizing the statistical independence of the data for conscious and unconscious conditions, ERSPs and ITPCs, respectively. But our current experiment found no significant correlation between vMORs and perceptual alternation in binocular rivalry after correcting for multiple tests. This result may suggest that theta phase alignment associated with the visual mismatch process plays an important role in the neural mechanism of APVA.

## Data availability statement

The raw data supporting the conclusions of this article will be made available by the authors, without undue reservation.

## Ethics statement

The studies involving human participants were reviewed and approved by The ethics committee of Tokyo University of Science. The patients/participants provided their written informed consent to participate in this study.

## Author contributions

YK, TU, and OA designed this study. YK collected and analyzed the data. YK drafted the manuscript and TU and OA edited and revised the manuscript. All authors contributed to the article and approved the submitted version.
